# Supplementary material for: Increasing Uptake of Maternal Pertussis Vaccinations through Funded Administration in Community Pharmacies
Source: Vaccines (Basel). 2022 Jan 20;10(2):150. doi: 10.3390/vaccines10020150 (PMC8876756; doi:10.3390/vaccines10020150)
Supplement: Supplementary file 1 [file vaccines-10-00150-s001.zip › EPI_208667_NZ_Quant_MS2_Supplemental_Material.pdf]

Supplementary Table S1. Description of Ministry of Health data sources and list of extracted variables

Data source	Extracted variables
National Maternity Collection (MAT)	
Contains information on each pregnancy that results in the delivery of a live born baby or a stillborn baby of at least 20 weeks' of gestation. The National Maternity Collection (MAT) provides statistical, demographic and clinical information about selected publicly-funded maternity services up to nine months before and three months after a birth.	NHI (encrypted)<br>Date of last menstrual period<br>Delivery date<br>Gestational Age<br>Maternal date of birth<br>Maternal ethnicity<br>Socioeconomic deprivation (NZDep13)[1]<br>District Health Board<br>Lead maternity carer at registration<br>Number of antenatal care visits
National Health Index (NHI) Database	
Contains demographic information for all healthcare users in New Zealand. Newborn infants have been registered in the database since 1992. This includes fetal deaths at more than 20 weeks' gestation and/or more than 400 grams birthweight. An individual's primary NHI number, date of birth, date of death and sex remain unchanged over time, but other fields may change.	NHI (encrypted)<br>Date of birth<br>Date of death<br>Sex<br>Maternal ethnicity<br>Socioeconomic deprivation (NZDep13)[1]
National Immunisation Register (NIR)	
Contains records of all immunisation events as per the New Zealand Childhood Immunisation Schedule. Children born from 2005 onwards have their immunisation enrolment and events recorded on the NIR. Immunisation events outside the National Immunisation Schedule, for example during pregnancy, are encouraged to be recorded but are not compulsory.	NHI (encrypted)<br>Date of vaccination<br>Vaccine<br>Provider
General Medical Subsidy Datamart (Proclaims)	
Proclaims contains the fee-for-service payments made to doctors for patient visits that have been processed by the HealthPAC Proclaim system	NHI (encrypted)<br>Date of vaccination<br>Vaccine
New Zealand Pharmaceutical Collection	
The Pharmaceutical Collection contains claim and payment information from pharmacists for subsidised community dispensings of prescribed medicines.	NHI (encrypted)<br>Date of dispensing<br>Therapeutic Group<br>Chemical Name

[1] NZDep13 combines nine variables from the 2013 New Zealand census which reflect eight dimensions of deprivation, including income, owning a house, access to a car. NZDep13 ranges from 1 (least deprived) to 10 (most deprived)

DRAFT

Supplementary Panel P1. Promotional activity

Promotional activity undertaken as part of the research in the intervention and control regions. The training was conducted before the research grants were in place, the remainder of the promotion occurred from April 2018 to September 2018 after the research grants were in place.

- A free evening training meeting covering maternal vaccinations for pharmacists in the main city in each of the three regions in late 2016 in the intervention area and in 2017 in the control areas.
- Two phone calls to each pharmacy. In each pharmacy on each occasion there was a conversation with the pharmacist most involved with vaccinations, or the main pharmacist in non-vaccinating pharmacies. At these phone calls, it was asked whether the pharmacy provided vaccinations or not. Messages were provided about maternal vaccinations, including suggestions to advise women collecting folic acid and iodine (usual pregnancy maternal supplements) to have maternal vaccines, and that pharmacies offering vaccinations connect with their local midwives. Email addresses were collected for pharmacies with social media pages to allow links to be sent to them for use on social media.
- Posters provided to all pharmacies to display about maternal vaccinations including availability in pharmacy and general practice.
- A small poster provided to all pharmacies for the pharmacy staffroom with key points on maternal pertussis and influenza vaccinations.
- Two quizzes aimed at frontline pharmacy staff about maternal vaccinations sent by fax to all pharmacies, with a prize draw for the correct answers.
- Twelve social media posts on Facebook, boosted to female consumers aged 20-55 years and later 15-65 years with keywords related to pregnancy. These messages were in the form of three brief videos promoting maternal vaccinations and noting their availability from pharmacies (intervention area for maternal pertussis and influenza, and control area for maternal influenza) and general practice (all areas). One of the videos was translated into Te Reo (language of Māori).
- Emails to vaccinating pharmacies with social media pages to link to new social media videos, and offering t-shirts promoting maternal vaccinations for pharmacy team members.
- Emails via the College of Midwives to their members notifying that pharmacy could administer free maternal influenza vaccination (all areas) and pertussis vaccination (Waikato DHB), as well being free in general practice, offering posters if desired, and providing a list of pharmacies in their region which provided vaccinations.
- A 3-minute maternal vaccination video for health care professionals shared via email of a link to pharmacies for which we had email addresses, sent also by the Pharmaceutical Society of New Zealand to their pharmacist members in the Waikato DHB region (virtually all pharmacists at that time). This video was also shared with midwives through the College of Midwives local branch contacts who were asked to email the link to their members with a reminder of maternal vaccinations being funded through pharmacies and general practice.

DRAFT

Supplementary Table S2. Demographics of study women at least 20 weeks' gestation between 1 November 2015 and 31 October 2019, by District Health Board of residence

	Total Cohort		District Health Board					
			Intervention Region		Control Regions			
			Waikato		Northland		Hawke's Bay	
	n	(%)	n	(%)	n	(%)	n	(%)
Total	27,576	(100.0)	15,329	(55.6)	6,359	(23.1)	5,888	(21.4)
**Prioritised Ethnicity**								
Māori	11,302	(41.0)	5,339	(34.8)	3,481	(54.7)	2,482	(42.2)
Pacific	1,137	(4.1)	643	(4.2)	146	(2.3)	348	(5.9)
Asian	2,889	(10.5)	2,080	(13.6)	371	(5.8)	438	(7.4)
Other	457	(1.7)	342	(2.2)	64	(1.0)	51	(0.9)
European	11,791	(42.8)	6,925	(45.2)	2,297	(36.1)	2,569	(43.6)
**Area Level Deprivation**								
Missing	1	(0.0)			1	(0.0)		
1	2,201	(8.0)	1,761	(11.5)	55	(0.9)	385	(6.5)
2	2,610	(9.5)	1,135	(7.4)	524	(8.2)	951	(16.2)
3	4,478	(16.2)	3,072	(20.0)	857	(13.5)	549	(9.3)
4	7,180	(26.0)	4,303	(28.1)	1,643	(25.8)	1,234	(21.0)
5	11,106	(40.3)	5,058	(33.0)	3,279	(51.6)	2,769	(47.0)
**Maternal age at LMP**								
12-19	1,948	(7.1)	980	(6.4)	519	(8.2)	449	(7.6)
20-24	5,458	(19.8)	2,899	(18.9)	1,371	(21.6)	1,188	(20.2)
25-29	8,268	(30.0)	4,690	(30.6)	1,844	(29.0)	1,734	(29.4)
30-34	7,498	(27.2)	4,336	(28.3)	1,599	(25.1)	1,563	(26.5)
35-39	3,508	(12.7)	1,941	(12.7)	802	(12.6)	765	(13.0)
40-45	869	(3.2)	468	(3.1)	219	(3.4)	182	(3.1)
46+	27	(0.1)	15	(0.1)	5	(0.1)	7	(0.1)
**Model of Care**								
DHB	322	(1.2)	1	(0.0)	198	(3.1)	123	(2.1)
GP	13	(0.0)	8	(0.1)	1	(0.0)	4	(0.1)
MWF	26,367	(95.6)	14,875	(97.0)	5,929	(93.2)	5,563	(94.5)
No LMC	817	(3.0)	405	(2.6)	216	(3.4)	196	(3.3)

	Total Cohort		District Health Board					
			**Intervention Region**		**Control Regions**			
			Waikato		*Northland*		*Hawke's Bay*	
	n	*(%)*	*n*	*(%)*	*n*	*(%)*	*n*	*(%)*
OBS	57	(0.2)	40	(0.3)	15	(0.2)	2	(0.0)
**Number of antenatal care visits**								
Missing	1,385	(5.0)	549	(3.6)	478	(7.5)	358	(6.1)
0-5	4,813	(17.5)	3,254	(21.2)	926	(14.6)	633	(10.8)
6-10	11,422	(41.4)	6,674	(43.5)	2,490	(39.2)	2,258	(38.3)
11-15	8,896	(32.3)	4,434	(28.9)	2,210	(34.8)	2,252	(38.2)
16-20	941	(3.4)	381	(2.5)	239	(3.8)	321	(5.5)
21+	119	(0.4)	37	(0.2)	16	(0.3)	66	(1.1)
**Parity of eligible pregnancy**								
Missing	863	(3.1)	407	(2.7)	230	(3.6)	226	(3.8)
0	11,556	(41.9)	6,640	(43.3)	2,489	(39.1)	2,427	(41.2)
1	7,597	(27.5)	4,332	(28.3)	1,656	(26.0)	1,609	(27.3)
2	4,122	(14.9)	2,224	(14.5)	1,031	(16.2)	867	(14.7)
3	1,844	(6.7)	962	(6.3)	476	(7.5)	406	(6.9)
4+	1,594	(5.8)	764	(5.0)	477	(7.5)	353	(6.0)
**Gestational Age (weeks)**								
28-38	7,974	(28.9)	4,396	(28.7)	1,729	(27.2)	1,849	(31.4)
39-41	18,702	(67.8)	10,294	(67.2)	4,478	(70.4)	3,930	(66.7)
42-43	900	(3.3)	639	(4.2)	152	(2.4)	109	(1.9)

LMP = last menstrual period; LMC = lead maternity carer
